# The Surgical Treatment of Cancer of the Rectum

**Published:** 1909-10

**Authors:** Cecil H. Leaf

**Affiliations:** Surgeon to the Cancer Hospital and to the Gordon Hospital for Rectal Diseases


### The Surgical Treatment of Cancer of the Rectum.

By CECIL H. LEAF, M. A., M. B. Cantab., F. R. C. S.
Surgeon to the Cancer Hospital and to the Gordon Hospital for Rectal Diseases.

(*The Polyclinic*, London, September, 1909.)

BEFORE considering the question of the surgical treatment of cancer of the rectum, we must briefly review some of the main anatomical points in connection with this part of the intestine.

The rectum—though its name implies straight—is by no means a straight tube, yet it is called so because it is less curved than any other part of the intestinal canal. It is usually divided into three parts: The first portion commences at the sacro-iliac synchrondrosis, usually of the left side, and extends to the lower part of the third piece of the sacrum, being directed towards the middle line. It is convex anteriorly and is 4½ inches in length. This portion of bowel may commence at the right sacro-iliac synchrondrosis. It is entirely covered with peritoneum, and, owing to the fact that it possesses a mesorectum, it has a certain amount of mobility. The second portion extends from the end of the third piece of the sacrum to the tip of the coccyx, and is concave anteriorly. This portion of bowel is only partially covered with peritoneum, its lower half being entirely destitute of it and its upper half only completely covered above. As you see in the diagram, the peritoneum comes lower down on the anterior than on the posterior surface, the distance of the anterior reflection being 3½ inches, and that of the posterior 5 inches from the anal orifice. The last segment of rectum is called the anal canal, and includes that portion of the bowel which is embraced by the levatores ani and sphincter muscles. It extends from the tip of the coccyx to the anal orifice and points somewhat backwards.

*The Blood Supply.*—About 1½ inch above the bifurcation of the aorta the inferior mesenteric artery arises. This vessel gives off the left colic and sigmoidean branches, and these two form a series of loops, from the convexity of which branches arise which supply the descending colon and sigmoid. These loops are situated about 1½ inch from the border of the intestine, and in certain operations, to which I shall refer later on, where the vitality of the sigmoid must be preserved, it is most important that these loops should not be interfered with, so that if the superior hemorrhoidal or sigmoid trunk has to be ligatured, blood can still flow into the loops through the branches of the left colic, and thus the vitality of the sigmoid is preserved. The two branches of the superior hemorrhoidal artery run downward on either side of the rectum, and lower down anastomose with the middle hemorrhoidal, which comes off, either directly from the anterior trunk of the internal iliac, or from the inferior vesical branch. The inferior hemorrhoidal comes off from the internal pudic, which, together with the sciatic artery, form the two terminal branches into which the anterior division of the internal iliac artery divides. The rectum derives by far its larger blood-supply from the superior hemorrhoidal, and, unless the trunk is secured in the abdomen, bleeding from the vessel may be troublesome and difficult to control during excision of the rectum. There are also other vessels which need a passing consideration. The middle sacral artery comes off, generally, from the aorta, immediately before its bifurcation and runs downward in the hollow of the sacrum. The two lateral sacral vessels come off on either side from the posterior division of the internal iliac and run towards the middle line and anastomose with lateral branches from the middle sacral. Twigs from all these vessels run in the loose fat behind the rectum and supply its posterior and lateral aspects, anastomosing with the hemorrhoidal arteries. During the removal of the coccyx, preparatory to excision of the rectum, the middle sacral artery is frequently divided and, as it is apt to retract, it may give rise to troublesome hemorrhage: also during the process of separating the second piece of the rectum from the hollow of the sacrum, twigs from this vessel and from the lateral sacral arteries may be ruptured and give rise to bleeding.

*The Nerves* which supply the bladder are derived from the sympathetic and from branches coming from the second, third, and fourth sacral nerves. Whether the musculature of the bladder derives its main nerve-supply from the sympathetic or directly from its sacral branches seems to be a moot question; but the practical point for us to remember is that if the third nerve-trunk is damaged on both sides, as may easily happen if Bardenhauer's incision (a modification of Kraske's operation, which consists in dividing the sacrum transversely at the level of the third sacral foramen) is carried out, then incontinence of urine will result owing to paralysis of the bladder thereby caused.

*The Lymphatics.*—To understand the lymph distribution of the rectum we divide the latter into three regions, which, as you will see in the diagram, do not correspond to the ordinary anatomical divisions of the rectum. These regions are (*a*) the lower, (*b*) the middle (*c*) the upper. The lower region (*a*) comprises the skin and subcutaneous region in the immediate vicinity of the anal canal, and overlying the external sphincter muscle. The lymph from this region drains into the superficial inguinal glands, and this area and the glands are colored blue in the diagram. (*b*) The middle area corresponds to the anal canal; the lymph from it passes into vessels which, roughly speaking, may be said to follow the course of the internal pudic and middle hemorrhoidal arteries, whence it eventually passes into glands situated along the inner side of the common iliac artery, and then into glands situated about the bifurcation of the aorta, and finally into glands situated on either side of the aorta. This district is colored brown in the diagram. (*c*) The upper region, which is colored yellow in the diagram, comprises the rest of the rectum, and includes what we have described as the first and second pieces of the rectum. The lymph from this region passes into some very small glands—the pararectal glands—embedded in the wall of the gut, and thence passes into vessels and glands which follow the course of the superior hemorrhoidal artery, and which are enclosed within the layers of peritoneum forming the mesorectum and mesosigmoid respectively. The lymph eventually passes into glands situated round the trunk of the inferior mesenteric artery, and thence drains into glands situated on either side of the aorta.

Before actually passing on to the question of the surgical treatment of cancer of the rectum, I should like to mention a few points in regard to the use of the sigmoidoscope. To examine patients with this instrument it is, of course, essential that the bowels should have previously been well cleared out. An anesthetic is not necessary, unless the patient is unduly nervous or apprehensive. The patient should lie in the right semiprone lateral position with the knees well bent and with a pillow under the buttocks. The instrument is first passed with the obturator in, but, as a rule, you will find that it cannot be pushed in more than 2½ or 3 inches without taking the obturator out and then commencing the inflation. The various curves which you see in this diagram will show you the different directions in which the instrument must be passed to enable it to enter the various segments of the rectum, but when once the obturator has been removed the actual "lie" of the mucous membrane over the end of the tube will always give an indication as to whether the end of the tube should be raised or lowered or turned to the front or back. I mentioned to you that the first part of the rectum joins the second part, either by inclining from the left or the right side to the middle line; consequently, therefore, when the sigmoidoscope is being passed from the second to the first part, with the patient lying in the right lateral semi-prone position, the handle of the instrument must either be depressed so as to tilt the tip of the instrument to the left side or *vice versa* as the case may be; and, as you will also see from the diagram, the handle of the instrument must be tilted somewhat backward so as to enable the tip to come well forward.

The entire rectum is 9½ inches in length, and the usual length of the sigmoidoscope is 12 3|8 inches, or 30 centimeters. When the whole length of the sigmoidoscope is passed, not only can the entire rectum be explored, but also rather more than 2 inches of the sigmoid as well. It must not be supposed that in every case the entire length of the instrument can be passed; indeed if the patient experience pain and discomfort it will be wise for the surgeon to recognise the fact that discretion is the better part of valor and make no attempt to pass the instrument its whole length, otherwise irreparable damage may be done. In many cases the tube can be pushed its whole length without difficulty, and in all cases where no obstruction exists it can be pushed in at least half its length, that is rather more than 6 inches.

We will now turn to the various operations which may be performed for cancer of the rectum, and then, as far as time will allow, discuss the relative advantages and disadvantages of each. First let us imagine we have a patient who has a growth which is in quite an early stage situated about 2½ inches above the anal orifice, which has not, we will imagine, involved the sphincter muscles or the lymphatic glands of the rectum. Unfortunately, it is only rarely that we come across such early cases, most patients not coming to the surgeon until the growth is much more advanced. However, if such a case presents itself, what is the operation that first suggests itself? Why, undoubtedly, to excise the affected portion and to do an end-to-end anastomosis. As you will see in the diagram, the part numbered 2 is removed and part No. 1 brought down and united to No. 3, the integrity of the sphincters being maintained. In those cases where the growth is situated a little lower down—that is, somewhat nearer the anal orifice—the operation may be modified in this way: The mucous membrane of the lower part of the anal canal having been removed, the lower portion of the upper segment (after that portion which bears the growth has been excised) is drawn through the anal canal, and its edges stitched to the margin of the anal orifice. Now what are the advantages and disadvantages of this operation? The advantages are: The peritoneum is not necessarily opened, thereby minimising the risk, the continuity of the bowel is maintained, and the sphincter action is preserved. What are the disadvantages? In that patients only rarely come to the surgeon in this early stage, this operation is only rarely applicable. Unless the peritoneum is divided there may be some difficulty in drawing down the upper segment of bowel, though there is no reason why the peritoneum should not be divided for this purpose. There may be tension on the sutures uniting the two ends which may result in their ultimately giving way. The operation is not a radical one in that the lymphatic glands are not removed to any extent, and in attempting to retain the action of the sphincter muscles, perhaps insufficient bowel below the growth is removed.

Let us now consider the treatment of cancers of the rectum which are more advanced and situated too high for removal by the perineal route and too low down for removal by the abdominal route. In order to obtain sufficient room to remove such growths, and knowing that the lower half of the sacrum and coccyx were not necessary to the integrity of the pelvis, Kraske, of Freiburg, proposed the removal of a portion of the sacrum up to the level of the third sacral vertebra. I will not here enumerate the various modifications of this operation which have from time to time been proposed, but need only say that to this operation as originally proposed there are many objections. In the first place there is the danger of damaging the third sacral nerve-roots and so of causing impairment of function or actual paralysis of the bladder. Again, chiefly owing to the teaching of Kocher, it is now recognised that there is no need to remove the lower portion of the sacrum, and that removal of the coccyx alone gives quite sufficient room. In Kraske's operation, modified as it now usually is, by removal of the coccyx only, the peritoneum is opened or not as required—usually it has to be opened; the rectum and part of the sigmoid is pulled down and the whole of the lower segment containing the growth is removed; the lower end of the upper segment of bowel which has been drawn down is now closed after the edges of the peritoneum have been united. If you will look at the diagram you will see these points diagrammatically represented, and you will also notice that a preliminary colostomy has been performed, a step which, in my opinion, should not be omitted in this particular kind of operation, as, of course, the artificial anus diverts the forces from the operation area, and, having been performed about a fortnight before the actual excision, it enables the bowel to be so much more thoroughly cleansed than could otherwise be the case. The objections to this operation are that, though an artificial anus has been made, the lower segment of bowel may form a *cul de sac* for the mucus which is secreted and may accumulate within it, and may give rise to a sinus which may take some time to close up. Again, the operation is not sufficiently radical, for although quite sufficient bowel both above and below the growth may be taken away, an insufficient amount of meso-rectum and mesosigmoid is removed in which glands already affected with cancer may be secretly lurking. Kraske himself now recognises this fact and is giving it up in favor of the abdomino-perineal or combined operation, in which the growth is attacked both through an abdominal and perineal incision. One of the first surgeons to perform the combined operation in this country was Miss Aldrich Blake. In this operation the abdomen is usually opened in the middle line, the sigmoid clamped and divided, and the divided lower end of the upper segment brought out through a small incision made to the left of the middle line; the mesosigmoid is divided along the line marked in the diagram until the superior hemorrhoidal trunk is reached, which is then ligatured. The upper end of the lower portion of sigmoid which had previously been ligatured is now separated as far as possible until the peritoneum forming the pelvic flow is reached, which is now divided round the bowel. During this stage care must be taken to avoid injuring the ureters, and any lymph glands felt enlarged round the common iliac vessels may be removed. The gut is now freed as much as possible from the structures in the front of and behind it, and is then tucked down underneath the peritoneum, the edges of which are then united over it. The abdominal wound is then sewed up. The patient is then turned into the right lateral and semiprone position, and after the necessary incision and separation of bowel has been carried out, the whole of the lower segment of gut containing the growth, including that part of the sigmoid with its contained mesosigmoid which has been tucked underneath the peritoneum, is removed. This is the operation very briefly described. What are its advantages? The superior hemorrhoidal artery which constitutes the chief blood-supply of the rectum is ligatured early, and so there is not much trouble afterward with the bleeding, for the middle and inferior hemorrhoidal arteries are easily ligatured as they are cut. A large amount of bowel can be removed, and, what is more important, a great deal of the mesosigmoid and mesorectum, in which, as I showed you, so many of the lymphatics of the rectum run, is removed also. The glands, too, along the course of the common iliac, and indeed right up to the bifurcation of the aorta, can be removed. In fact, "the zone of the upward spread" of cancer, as Miles[1] puts it, is removed. What objections can be urged against this operation and how may these objections be answered? The mortality is high, being given by Swinford Edwards[2] as 40.9 and Miles[1] as 4.16 per cent., but, as the latter points out, cancer of the rectum is almost invariable fatal, and the mortality from the combined operation, though undoubtedly high at the present time, will in the future, with an improved technic, be undoubtedly lessened. The mere fact of having to divide the bowel, having to separate the rectum from surrounding structures, to which its walls may be matted by inflammation or by invasion of the growth, are elements favoring the onset of peritonitis. Again, it may be necessary to make rather a wide division of the peritoneal pelvic floor, and it may not be possible to bring the edges together again without causing some tension. Should any of the sutures pull through, a rent may be caused through which a coil of small intestine may become prolapsed

1. Lancet, London, 1908, xi, p. 1812, "A Method of Performing Abdomino-Perineal Excision for Carcinoma of the Rectum and the Terminal Portion of the Pelvic Colon," by W. Ernest Miles, F.R.C.S.

1. Ibid.

2. "Cancer of the Rectum," by F. Swinford Edwards, F.R.C.S.

and strangulated. Many patients are old and are not likely to stand the shock of such an operation. In these cases it is best to do the operation in two stages, as recommended by Arbuthnot Lane, and if shock is present it can always be met either by infusion of saline into the axilla, as recommended by Lane, or directly into a vein. It may be said that as the abdomen has to be opened the gravity of the operation is thereby increased; but, as a matter of fact, there are several advantages in opening the abdomen. In the first place, it gives the surgeon an opportunity of estimating how far the disease has progressed, and whether the case is really an operable one or not. Drew[3] found that in 8 per cent. of his cases, circumstances were present which entirely vetoed an operation, and Funke, quoted by Drew, found that in two-thirds of his cases that were operated upon, the glands were involved—two additional reasons surely for opening the abdomen. Again, it has been urged against this operation that a permanent colostomy is formed, through which the patient must always discharge his feces and over which he has no control. That the patient does have a permanent colostomy cannot be denied, but with a suitable apparatus this should not prove a serious disadvantage, and patients can soon learn to arrange their toilet and go about in comfort. Ryall[4] has lately devised a new method by which patients may secure a certain amount of control. The method briefly consists in making the colostomy through the rectus abdominis muscle, which is incised longitudinally; some of the deeper muscular fibers are separated on either side of the incision, and one strand is looped over the other, and the gut brought out through the loop thus formed which thus encircles the gut. The cut edges of the bowel are sutured to the skin. A double sphincter action is thus formed, the longitudinal fibers being those formed by the superficial portion of the rectus, and the circular those formed by the loop of the deeper portion. Another variety of the combined operation is what may be termed the invagination method, which consists essentially in invaginating one part of the bowel into another, and then bringing the whole of the invaginated portion, including the growth outside the anal canal, and then removing the portion of bowel containing the growth and doing an end-to-end anastomosis. The two diagrams illustrate this method of operating, and you will easily understand that this method is only applicable when an invagination can be thus artificially produced. This operation has been

---

3. Brit. Med. Journ., Lond., 1908, xi, p. 990, "On the Selection of Method for Operating on Cancer of the Rectum," by Douglas Drew, F.R.C.S.

4. Lancet, July 3, 1909, "A New Method for Attempting to Secure Sphincteric Control after Colostomy," by Charles Ryall, F.R.C.S.

successfully performed by Barker,[5] who points out the necessity of maintaining the vitality of the sigmoid by not interfering with its vascular anastomotic loops, and also by Aslett Baldwin. One of the great difficulties of the operation consists in producing the invagination, and one of its great advantages is the fact that the anastomosis is made entirely outside the abdomen.

The tendency for the surgical treatment of cancer in other regions is to make the operations more radical, and, I think, rightly so; and so in the surgical treatment of cancer of the rectum I think there is little doubt that in future we shall find that the majority of cases, if not too far advanced for a radical operation, are best dealt with by some form of combined operation.

